# Facebook Support Groups for Rare Pediatric Diseases: Quantitative Analysis

**DOI:** 10.2196/21694

**Published:** 2020-11-19

**Authors:** Sarah Catrin Titgemeyer, Christian Patrick Schaaf

**Affiliations:** 1 University of Cologne Cologne Germany; 2 Institute of Human Genetics Heidelberg University Heidelberg Germany; 3 Institute of Human Genetics University Hospital Cologne Cologne Germany

**Keywords:** pediatric rare diseases, rare diseases, support group, online support, Facebook support group, social media, parent support, support group privacy, counseling

## Abstract

**Background:**

Loneliness, social isolation, and feeling disconnected from society are commonly experienced by parents of children with rare diseases and are, among others, important reasons for special supportive care needs. Social networking platforms are increasingly used for health communication, information exchange, and support. In the field of rare pediatric diseases, qualitative studies have shown that Facebook online support groups are utilized by and beneficial for persons affected by rare pediatric diseases. Nonetheless, the extent of this usage has not been investigated.

**Objective:**

This study aims to provide a comprehensive quantitative analysis of the extent of Facebook usage as a tool for rare pediatric disease support groups and to explore factors that influence a disease’s representation on Facebook. These results potentially offer important insights for future public health initiatives and give direction to further research that can give much needed support to parents of children with rare diseases.

**Methods:**

We determined rare pediatric diseases using the inventory of the online portal Orphanet. Facebook support groups were identified by searching 5 synonymous disease descriptions using the group category search bar. Disease- and group-describing parameters were statistically analyzed using standard descriptive statistical methods.

**Results:**

6398 Facebook support groups, representing 826 diseases (19.5% of all searched diseases), were found. 69% are private groups. Group type, size, activity (sum of posts, comments, and reactions calculated by Facebook), new memberships, and language varied largely between groups (member count: minimum 1, maximum 23,414; activity last 30 days: minimum 0, maximum 3606). The highest percentage of awareness and information groups was found for teratogenic diseases (18/68, 26%). The odds of finding a Facebook group increased according to the level of information available about the disease: known prevalence (odds ratio [OR] 3.98, 95% CI 3.39-4.66, *P*<.001), known disease type (OR 3.15, 95% CI 2.70-3.68, *P*<.001), and known inheritance mode (OR 2.06, 95% CI 1.68-2.52, *P*<.001) were all associated with higher odds of finding a Facebook group, as was dominant compared to nondominant inheritance (OR 2.05, 95% CI 1.74-3.42, *P*<.001). The number of groups per disease increased with higher prevalence.

**Conclusions:**

Facebook is widely used as a tool for support groups for rare pediatric diseases and continues to be relevant. Two-thirds of the groups are private groups, indicating group participants’ need for privacy, which should be further explored. The advantages and limitations of Facebook as a tool for support groups in the field of rare diseases should be further investigated as it will allow health professionals to use Facebook more meaningfully in their counseling and guidance of affected individuals and their family members.

## Introduction

### Background

Many parents of children affected by rare diseases described caring for a child with a rare disease to be highly isolating—with loneliness, social isolation and feeling disconnected from society being mentioned as common problems [1]. Most parents had never come into contact with other parents of a child with a similar condition to that of their own child, and many were dissatisfied with the overall support that they had received for their child with a rare disease from any source [1]. Since rare diseases have per definition a very low prevalence (European definition of rare diseases: <1 per 2000) [2], affected individuals are often geographically dispersed. For many rare diseases there is a deficit of medical and scientific knowledge [2]. Rare diseases are often serious, chronic, and progressive, and persons affected by rare diseases are more psychologically, socially, economically, and culturally vulnerable [2]. Hence, parents of children with rare diseases have special supportive care needs [1].

Parent-to-parent peer support has been shown to have beneficial effects on parents of children with disabilities and children with additional needs [3]. Parents benefit from support groups most importantly by building social connections, gaining a sense of belonging [4,5], and developing a sense of control [5]. Support groups provide an environment for parent-to-parent support, which offers several benefits through improved social support. Participating parents can experience improved social connections [4], a heightened sense of control [5], higher family life congruence [6], and lower consequences of perceived stress [7].

Social networking platforms are increasingly used for health communication, information exchange, and support. Benefits of using social media for health-related online communication and community include connectedness, increased community support, and online support groups [8]. Several advantages of social media for online support have been identified, including international scope, unlimited number of participants, cost-effectiveness [9], and 24-hour availability [10]. However, there are limitations, such as questionable reliability [11], accuracy [9], quality [12], application to personal situations [11], and the possible misinterpretation [9] of information found online and on social media.

Founded in 2004, Facebook is one of the longest existing social networking platforms [13]. In the second quarter of 2019, Facebook reported 2.41 billion monthly active users [14]. Thus, chances are presumably high that another person affected by the same disorder also uses Facebook and would be eligible to form a support group. Facebook allows persons to connect independent of geographic location and offers options for both individual and group communication [13].

### Prior Work

Parents of children with rare diseases are active internet users, search for information online [15], and use social media such as Facebook to communicate and link with others [12], showing that most parents are already familiar with Facebook and are, therefore, likely have the required social networking skills. Therefore, they could benefit by extending their Facebook usage to participation in support groups quite effortlessly.

To our knowledge, only little research exploring the specific topic of online support groups for rare pediatric diseases has been conducted so far. Content analyses of specific online and Facebook support groups have been performed (eg, on groups for cleft lip and palate, clubfoot, Hirschsprung disease, autism spectrum disorders, Dravet syndrome, and related epilepsy disorders [16-21]). Group members benefit from giving and receiving informational and emotional support and from connecting with others since meeting others with similar experiences has been shown to decrease isolation [10,16-19].

### Research Rationale

Social support can provide several benefits for parents caring for children with chronic diseases, disabilities, additional needs, behavioral problems, and rare diseases [3-10,12,13,15,16,18,20]. Qualitative studies have shown that Facebook is utilized by and beneficial for persons affected by rare pediatric diseases. However, since these studies focused on specific conditions or groups, they failed to reflect the extent of support group usage and the overall representation of rare pediatric diseases on Facebook.

Our study therefore aimed to provide a comprehensive quantitative analysis of the extent of Facebook usage as a tool for rare pediatric disease support groups and at analyzing disease- and group-describing parameters to explore factors that influence a disease’s representation on Facebook.

These results may offer important insights for future public health initiatives and give direction to further research which can improve much needed support of parents of children with rare diseases. The analysis of Facebook groups dedicated to rare pediatric diseases and their development over time, for example, shows how many groups and individuals could benefit from an optimization of support groups conditions on Facebook. Initiatives that aim to promote communication among affected families can use this analysis to learn about support group structures such as group sizes and privacy settings. Having built the foundation of a quantitative analysis, future research can, for example, focus on a more in-depth qualitative analysis of Facebook group. Furthermore, this study points to the need for health professionals who treat individuals with rare pediatric disorders or affected parents or caregivers or provide genetic counseling to get better acquainted with the topic of social media support groups in order to understand and promote the communication among parents or caregivers of children with rare disorders.

## Methods

### Data Collection

Rare diseases with childhood manifestation were identified using the inventory of the online portal for rare diseases and orphan drugs Orphanet). Orphanet uses the European definition of rare disease [22]. A rare pediatric disease is defined as a disease with onset before adulthood; thus, age of onset had to be defined as antenatal, conatal/neonatal, infancy, childhood or adolescent but not adult, older adult, or all ages. Data collected included disease name, 4 synonyms, ORPHAcode, Online Mendelian Inheritance in Man number, International Statistical Classification of Diseases Tenth Revision, disease prevalence, inheritance mode (autosomal or sex-linked, recessive or dominant, etc), age of onset and disease type (monogenic, deletion or alteration of a single gene; chromosomal, alteration in the number or structure of a chromosome; microdeletion, deletion of a small chromosomal segment; teratogenic disorder, result of exposure to teratogenic agent; mitochondrial; infectious disease; multigenic or multifactorial). Age of onset and disease type information was extracted from disease name or Orphanet disease description. Data were collected between January 1, 2019 and March 13, 2019.

Facebook support groups were identified by searching 5 synonymous disease names or descriptions using the Facebook group category search bar. The researcher used a Facebook account, newly created for this purpose, that contained only the researcher’s name, picture, gender (female) and location (Cologne, Germany); but no activity (likes, shares, etc) expect for searching for aforementioned disease names. Groups were subcategorized according to their specific focus using the information available from the group title, the group category provided by Facebook’s group categorization or the publicly available group description. Groups had to be clearly recognizable as support groups, groups to raise awareness and information, or support groups for individual patients. Groups that explicitly focused on research, fundraising and charity, medication sales, and disease-related pages were excluded from analysis (examples for categorization using group title *Disorder A fundraiser* was categorized as focus on fundraising and excluded from analysis, *Child B’s journey with disorder C* was categorized as personal support group, *Disorder D: spread awareness* was categorized as awareness and information group). Data collected on Facebook included group type, name, language, privacy setting (public or private), foundation date, member count, and group insights. Group insights are provided by Facebook and displayed on each group information page, regardless of privacy status. They report activity (sum of posts, comments, and reactions calculated by Facebook) and new members during the past 30 days. Only groups with at least 1 member qualified. Foundation dates for groups on Facebook can be entered automatically or manually; manually entered foundation dates before Facebook launch in 2004 were excluded from analysis due to a lack of reliability of information. Data were collected between March 13, 2019 and March 31, 2019.

### Data Analysis

Data were analyzed using standard descriptive statistical methods using SPSS statistics (version 26; IBM Corp). Normally distributed data are presented using mean and standard deviation, skewed distributions are presented using median and interquartile range, binary and categorical variables are presented using counts and percentages. Odds ratios (ORs) were calculated. The 1-sample Kolmogorov-Smirnov test was applied to test for normal distribution. Nonparametric tests (Spearman ρ correlation, independent sample Mann-Whitney *U* test) were applied. Binomial and chi-square tests were used to investigate binary and categorical variables.

This study has been reviewed by the Ethics Commission of the Medical Faculty of the University of Cologne (protocol 19-1027), and all research has been carried out within the scope of the approval.

## Results

### General

A total of 4246 rare disorders with onset before adulthood were identified using the Orphanet inventory, and 6398 support groups were found on Facebook. These groups represent 826 diseases, which amounts to 19.5% of all searched diseases.

The 10 diseases for which the most Facebook support groups were found are Down syndrome (145/6398, 2.3%), 22q11.2 deletion syndrome (117/6398, 1.8%), hypoplastic left heart syndrome (117/6398, 1.8%), Turner syndrome (97/6398, 1.5%), gastroschisis (95/6398, 1.5%), cleft lip and palate (93/6398, 1.5%), Ehlers-Danlos syndrome (93/6398, 1.5%), craniosynostosis (92/6398, 1.4%), microtia (90/6398, 1.4%), and retinoblastoma (89/6398, 1.4%). An alphabetic list of diseases on Facebook, including Online Mendelian Inheritance in Man number and the respective number of groups, is presented in Multimedia Appendix 1.

**Figure 1:**
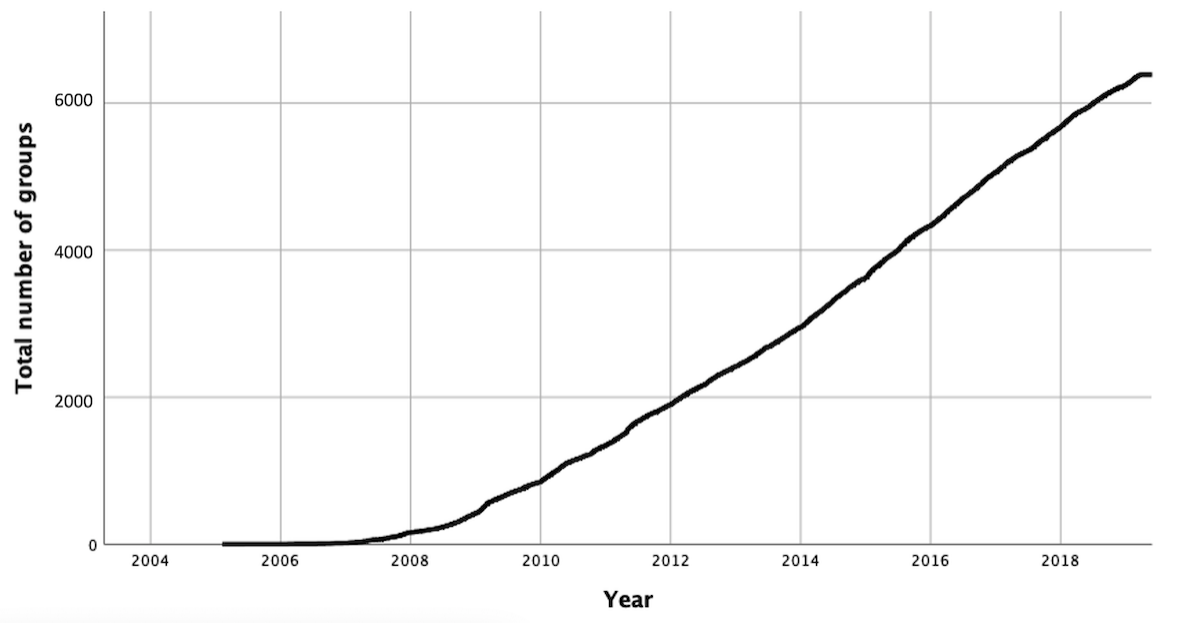
Development of the total number of Facebook support groups for pediatric rare diseases over time.

The total number of Facebook groups has continuously increased since 2008 (Figure 1). The number of newly created Facebook support groups shows fluctuation with an overall increase (Multimedia Appendix 2).

### Disease-Describing Parameters

Of the 4246 diseases listed on Orphanet, 529 (12.9%) diseases show antenatal and 2815 (68.9%) show conatal/neonatal onset, 2167 (53.0%) start during infancy, 1074 (26.3%) during childhood, and 165 (4.0%) show adolescent onset. More than one age of onset may apply.

Table 1 depicts the distribution of disease type and prevalence among all identified diseases and diseases with at least 1 support group and the number and percentage of Facebook support groups per disease type and prevalence. 274/934 (29.3%) of the monogenic diseases, 51/145 (35.2%) of the chromosomal, 85/158 (53.8%) of the multigenic or multifactorial, and 4/22 (18.2%) of the teratogenic diseases found on Orphanet are represented on Facebook, and 376/2830 (13.3%) of the diseases with unknown disease type. The mean number of groups per disease increases with increasing prevalence (mean 2 for prevalence <1 per 1,000,000; mean 5 for prevalence 1-9 per 1,000,000; mean 14 for prevalence 1-9 per 100,000; mean 28 for prevalence 1-9 per 10,000).

Table 2 displays the number and percentage of diseases on Orphanet and diseases with at least one group following the different inheritance modes. More than one inheritance mode may apply.

ORs were calculated to compare the probability of a disease with known or unknown disease-describing parameters to be represented by at least one Facebook group: known prevalence (OR 3.98, 95% CI 3.39-4.66, *P*<.001), known disease type (OR 3.15, 95% CI 2.70-3.68, *P*<.001), and known inheritance mode (OR 2.06, 95% CI 1.68-2.52, *P*<.001) are all associated with higher odds of finding a Facebook group; as is dominant compared to nondominant inheritance (OR 2.05, 95% CI 1.74-3.42, *P*<.001).

**Table 1:** Diseases and Facebook support groups by prevalence and type of disease.

		Diseases found on Orphanet (n=4246), n (%)	Facebook support groups (n=6398), n (%)	Diseases with ≥1 group (n=826), n (%)
**Prevalence**				
	<1 per 1,000,000	2516 (59.3)	620 (9.7)	253 (30.6)
	1-9 per 1,000,000	150 (3.5)	452 (7.1)	85 (10.3)
	1-9 per 100,000	188 (4.4)	1825 (28.5)	132 (16.0)
	1-9 per 10,000	77 (1.8)	1251 (19.6)	44 (5.3)
	1-9 per 1000	1 (<0.1)	0 (0.0)	0 (0.0)
	Unknown	1314 (30.9)	2250 (35.2)	312 (37.8)
**Disease type**				
	Monogenic	934 (22.0)	1623 (25.4)	274 (33.2)
	Chromosomal	145 (3.4)	655 (10.2)	51 (6.2)
	Mitochondrial	25 (0.6)	11 (0.2)	2 (0.4)
	Infectious disease	2 (<0.1)	1 (<0.1)	1 (0.1)
	Multigenic or multifactorial	158 (3.7)	988 (15.4)	85 (10.3)
	Teratogenic disorder or infectious fetopathy	22 (0.5)	68 (1.1)	4 (0.5)
	Micro-/contiguous gene deletion/duplication/triplication	130 (3.0)	290 (4.5)	33 (4.0)
	Unclassified	2830 (66.7)	2762 (43.2)	376 (45.5)

**Table 2:** Pediatric rare diseases on Orphanet and diseases with at least one group that follow the different inheritance modes (more than one may apply).

Type^a^	Pediatric rare diseases on Orphanet that follow this inheritance mode, n (%)	Pediatric rare diseases with ≤1 group that follow this inheritance mode, n (%)
Autosomal recessive	1765 (55.6)	314 (38.0)
Autosomal dominant	971 (30.6)	279 (33.8)
X-linked recessive	306 (9.6)	75 (9.1)
X-linked dominant	70 (2.2)	25 (3.0)
Multigenic or multifactorial	74 (2.3)	31 (3.8)
Mitochondrial	15 (0.5)	1 (0.1)
Y-linked	1 (<0.01)	0 (0.0)
Inheritance not applicable	513 (16.2)	197 (23.8)

^a^More than one may apply.

### Group-Describing Parameters

#### Group Type

The support groups are further divided into the following subcategories: general support groups (4385/6398, 68.5%), personal support groups (828/6398, 12.9%), support groups with focus on awareness and information (450/6398, 7.0%), not further specified groups (649/6398, 10.1%), groups for several diseases (86/6398, 1.3%). The following were not included: main focus on research (n=147), fundraising and charity (n=338), and medication sales (n=4). The highest percentage of awareness and information groups was found for teratogenic diseases (18/68, 26%).

#### Group Language

Disease names or synonyms were entered into the Facebook search bar in English. Groups were mostly English speaking (5721/6398, 89.4%), with a smaller number listing French (227/6398, 3.5%), Spanish (99/6398, 1.5%), German, Dutch, Portuguese, Swedish, Turkish, Polish, or Danish as the group language. In total, 38 different group languages were found (Multimedia Appendix 3).

#### Group Statistics

Group-describing parameters were not normally distributed. The sum of group members in all groups amounted to 1,784,435. Membership in more than one group was possible. The median number of members was 33 (IQR 183, Q1 5, Q3 188; for comparison: mean 278.91, SD 989.46). This varied between personal support groups (mean 87.50, IQR 238.75), general support groups (mean 44, IQR 217), and awareness and information groups (mean 15, IQR 105.25). The maximum group member count was 23,414 in a group for pediatric multiple sclerosis, the minimum was 1 member in 496 groups. Of these, 326 groups were the only Facebook support group for the respective disease. Of these groups, 268 (82.2%) were general support groups, 12 (3.7%) were personal support groups, 11 (3.4%) were awareness and information groups, 5 (1.5%) were groups for several diseases, and 30 (9.2%) were not further specified groups.

Throughout all groups, 84,966 new posts, comments and reactions were found (range 3606, minimum 0, maximum 3606; mean 0, IQR 3, Q1 0, Q3 3). In total, 4021 groups showed no group activity. 35,119 persons joined the identified Facebook support groups (range 1357, minimum 0, maximum 1357; mean 0, IQR 1, Q1 0, Q3 1).

### Privacy Settings

When set to private, content, such as posts and pictures, is only accessible to members whose membership must be approved by a group administrator. Group title, group description, and group statistics including member count, new members last 30 days and activity last 30 days are always publicly available. Of the identified groups, 69% (4414/6398) are private, and 31% (1984/6398) are public. The sum of group members was 1,468,102 in private and 316,333 in public groups, with a maximum of 23,414 members in a private and 17,000 members in a public group. The median member count was higher in private (mean 46) than in public groups (mean 14). The median activity and new members in the 30 days prior to analysis was 0 for both private and public groups, with a sum of 78,023 activities in private and 6943 activities in public groups and a sum of 29,566 new members in private and 5553 new members in public groups. Performing an independent-sample Mann-Whitney *U* test showed that the distribution of the group-describing parameters differed slightly between the 2 privacy settings with higher member count, activities last 30 days, and new members last 30 days in private groups (member count: *U*=5,296,374, *z*=13.44, *P*<.001, effect size *r*=0.17; activities last 30 days: *U*=5,602,193, *z*=20.65, *P*<.001, effect size *r*=0.29; new members last 30 days: *U*=5,104,178, *z*=13.04, *P*<.001, effect size *r*=0.16).

### Correlation Analyses of Group- and Disease-Describing Parameter

Correlation analyses showing relations between group- and disease-describing parameters are displayed in Table 3. The minimal age of onset correlates neither with the number of groups per disease nor with the group member count.

**Table 3:** Spearman correlations between disease- and group-describing parameters.

Interpretation, variables		ρ	*P* value
**Significant strong positive correlation**			
	Recent group activity and number of new members	0.769	<.001
**Significant moderate positive correlation**			
	Prevalence and number of groups per disease	0.530	<.001
	Group member count and recent group activity	0.691	<.001
	Group member count and new group members	0.628	<.001
**Significant weak positive correlation**			
	Prevalence and group member count	0.101	<.001
	Time that a group exists and member count	0.111	<.001
**No significant correlation**			
	Time that a group exists and recent group activity	0.011	.39
	Time that a group exists and number of new members	–0.002	.85
	Disease’s minimal age of onset and number of groups per disease	–0.021	.55
	Disease’s minimal age of onset and group member count	0.006	.62

## Discussion

### Principal Findings

Facebook is widely used as a tool for support groups for individuals affected by rare pediatric diseases. This study has shown that, for approximately every fifth rare pediatric disease, one can find an existing Facebook support group. Group type, size, activity, new memberships, privacy settings, and language vary largely between groups.

Within the first years after the launch of Facebook in 2004, only a few Facebook support groups for rare pediatric diseases were created. Starting 2008 and onward, the total number of Facebook groups has been following almost linear growth. Consequently, we expect the number of groups and the number of diseases represented on Facebook to further increase in the coming years.

### Facebook Support Group Subtypes

Support group subcategories allow different group focus and benefits. Analyses of some general support groups have shown that group members give and receive informational and emotional support [19], exchange knowledge and advice [18], and benefit from the ability to connect with others via Facebook [16].

Personal support groups (about every eighth identified group) are dedicated to one specific child with a certain disorder. Information about this child’s health is shared and discussed. This group format has similarities with a blog but offers more personal two-way communication and thereby opportunities for emotional support. These groups’ creator and members may especially benefit from having a place to speak openly about the disease and feelings as well as from receiving emotional support, which are 2 main benefit categories identified by White and Dorman [9]. A possible explanation for why these groups show the highest median member count could be a different target group. While other support groups are usually joined by parents and other immediate family members [12,16-18,20], these groups are probably also joined by family friends, who receive health updates and offer comfort, but who do not have a child with a similar condition. It could be of interest to evaluate the impact of this method of receiving social support in the context of rare diseases, since such groups do not depend on disease prevalence and finding others with the same condition.

Almost every tenth identified support group also focuses specifically on creating awareness and providing information. This is in agreement with previous studies’ findings: families of patients with rare disease often become involved in raising public awareness [10], and social media can increase rare disease awareness [23]. The highest percentage of awareness and information groups was found for teratogenic diseases. Many teratogenic diseases are preventable disorders and parents, caregiver, or patients might therefore utilize Facebook groups to spread awareness to prevent future cases of the same disease.

### Insights Gained From Group Statistics

#### Facebook Group Accessibility

Even though we used English search terms, we found support groups in 38 different languages, indicating that Facebook support groups are a worldwide development. This supports that Facebook is a fitting tool for support groups since it is a globally accessible platform [13]. Facebook offers the possibility to easily and inexpensively share information 24 hours a day and time-zone independent [10,21]. Parents of children with rare diseases are active internet users and use social media such as Facebook [12,15]. Together with the aspect of internationality, Facebook is therefore accessible as a tool for support groups for many, if not most, caregivers for a child with a rare disease.

#### Group Members and Activity

Group sizes vary greatly. Group member count can be influenced by multiple factors, as we have shown for disease prevalence. Other factors may include group promotion among affected families and by health professionals.

Correlation analyses showed that groups with more group members also had slightly more recent group activity and new members. Support group participants could therefore benefit from joining a larger Facebook group, since it offers more active discussions and more individuals to connect with. Group activity and new member count showed a strong positive correlation, which indicates that new members start new conversations.

Many groups did not show any group activity. This could be either coincidental or indicate that these groups are inactive. Even if groups are formally inactive, there might still be private conversations between group members using Facebook Messenger or other personal messaging services, which we were not able to evaluate.

#### Looking for Others to Start a Support Group

Our analysis showed that 326 persons created a group for a specific disease, but at the time of study no one had joined their group, which means that they were unsuccessfully looking for someone to start a support group with. Possible reasons are that no one directly or indirectly affected by the same condition has turned to Facebook in order to look for a support group, or that no one is available for a support group on Facebook. Regarding the first explanation, it could be helpful to raise awareness of Facebook as a tool for rare pediatric disease support groups among parents and caregivers. A survey of caregivers of children with Autism Spectrum Disorders found that caregivers whose diagnosing clinician had referred them to a support group were more likely support group participants [20]. Therefore, health professionals ought to get better acquainted with the topic of social media support groups if they aim to promote the communication among parents and caregivers of children with rare disorders.

#### Facebook Support Group Privacy

A group’s privacy setting limits access to the group. When set to private, membership must be validated by a group administrator before content such as posts and pictures can be accessed or created. Group description and group statistics are publicly available. Our analysis showed that two thirds of the groups were private, which is in agreement with a survey conducted among patients with newly described or rare genetic findings, of whom 60% were uncomfortable with sharing information in a public group [13].

Because of the need of a validation before joining a private group, we expected public groups to have higher member counts, but private groups’ median member count resulted to be three times higher. More individuals appear to prefer joining private support groups, which indicates members’ preference of a more private environment when sharing experiences related to their children’s health.

Nevertheless, sharing information in a private Facebook group still means sharing information about a child online and oftentimes with (relative) strangers. Confidentiality and privacy issues are an important topic, since group participants are often unaware of risks of disclosing personal information [11]. Studies on mothers’ habits of sharing private details on their children on Facebook regardless their children’s health status have shown that mothers become increasingly aware of privacy issues on Facebook and try to find a balance between the need for privacy and the benefits of openness; some felt that some information was not appropriate to share [24,25]. This topic is particularly challenging since children cannot object to sharing information and pictures online, but might experience negative consequences later in life. Privacy issues therefore need to be investigated in the context of Facebook as a tool for rare pediatric disease support groups.

#### Factors That Influence a Disease’s Representation on Facebook

The more individuals are affected by a disease, the more individuals potentially turn to Facebook to look for or create a support group. Many diseases with higher prevalence have several support groups, and these groups’ descriptions often include geographic locations, eg different countries or states. Facebook group members have been shown to organize meetings for particular events [19], and Facebook groups organized according to members’ locations could facilitate this.

The analysis of variables collected in this research showed that the odds of finding a Facebook group for a disease with known prevalence are almost four times higher compared to with unknown prevalence, for a disease with known inheritance two times higher than with unknown inheritance, and for a disease with known disease type more than three times higher than with unknown disease type. These findings suggest that the chances of finding a Facebook group increase with a higher level of understanding about the disease. New diseases are described regularly, but it takes time and resources to investigate newly described diseases, and for many rare diseases there is a subsequent deficit of medical and scientific knowledge [2]. It also takes time until more affected children have been diagnosed. This limited information and the factor of time influence the chance of finding a group on Facebook. Other factors may play a role as well. The impact of disease-specific mortality, for example, could be of great importance and might therefore be of interest for further investigation.

The odds of finding a Facebook support group for a disease with possible dominant inheritance are twice as high as for a disease without dominant inheritance. The risk of transmission in dominant diseases is 50%. Individuals affected by dominant diseases therefore encounter several challenges, such as reproductive decision making, feelings of guilt, and the need to communicate genetic risk with their children and family members [26]. These challenges can influence an individual’s need for peer support, which may, in part, explain the higher probability of finding groups for dominant diseases.

#### Consequences for Treating Physicians and Other Health Care Professionals

This study indicates a need for health care professionals to become acquainted with social media as a tool for support groups, since it is already widely used. To allow informed decision making on whether to refer parents and caregivers of children with a rare disease to Facebook, more research about the strengths and limitations of Facebook as a tool for support groups is needed. If treating physicians decide to promote Facebook support groups, they can inform patients about the chances of finding a group, which is 1 in 5 overall but higher for monogenic, chromosomal, and multigenic or multifactorial diseases. Physicians with a focus on certain diseases can use our research to explore the extent of Facebook usage for groups for the respective disease. Furthermore, this study strengthens the importance of further research about rare diseases, since knowledge about a rare disease also influences the availability of support groups.

### Study Strengths and Limitations

This study is a broad-scope analysis. All Orphanet-listed diseases which conform to the inclusion criteria were searched on Facebook using 5 synonymous disease descriptions. Nevertheless, it is impossible to know whether all existing support groups have been identified. Even though the Facebook search was conducted in English, groups in 38 different languages were identified. It is possible that not all existing non-English groups have been found, especially when disease names differ largely from the English disease name. Other groups regardless of group language may not at all use a disease description in their group title. Facebook search engine optimization may have influenced our search results using the researcher’s information regarding location and gender. To minimize this effect, no other activities such as viewing, liking or sharing have been performed. Group activity and the number of new memberships could vary and since this was a cross-sectional study our data may not be representative. This could be investigated by repeating the study at another point in time. The limited information on Orphanet also limited our analysis. Disease type was evident for only a third of the diseases and information on prevalence was provided for only two-thirds of the diseases.

### Conclusion

There has been a continuous rise in the number of support groups and diseases represented on Facebook since 2008. We expect that the relevance of Facebook as a tool for rare pediatric disease support groups will continue to increase. Group type, size, activity, new memberships, privacy settings, and language vary largely between groups. Support group subcategories allow different group focus. The odds of finding a Facebook group have been shown to increase according to the level of information available about the disease, and the number of groups increases with higher prevalence. Two-thirds of the groups are private groups, indicating the group participants’ need for privacy, which should be further explored. More research is necessary to investigate the strengths and limitations of Facebook as a tool for support groups in the field of rare disease. This will allow health professionals to use Facebook more meaningfully in their counseling and guidance of affected individuals and their family members. It may also allow Facebook and other similar social media platforms to improve their toolkits and offerings for individuals affected by rare diseases.

## Appendix

Multimedia Appendix 1 Alphabetic list of diseases.

Multimedia Appendix 2 The number of newly created Facebook support groups shows fluctuation with an overall increase.

Multimedia Appendix 3 Full list of Facebook support groups by language.
