# Supplementary material for: Facebook Support Groups for Rare Pediatric Diseases: Quantitative Analysis
Source: JMIR Pediatr Parent. 2020 Nov 19;3(2):e21694. doi: 10.2196/21694 (PMC7714646; doi:10.2196/21694)
Supplement: Multimedia Appendix 1 [file pediatrics_v3i2e21694_app1.doc]

Multimedia Appendix 1. Alphabetic list of diseases represented on Facebook including ORPHAcode, OMIM number and number of groups per disease.

| **Disease name** | **ORPHAcode** | **OMIM number (1)** | **OMIM number (2)** | **number of groups** |
| --- | --- | --- | --- | --- |
| 15q overgrowth syndrome | 314585 | 614846 | - | 1 |
| 15q11q13 microduplication syndrome | 238446 | 608636 | - | 1 |
| 15q13.3 microdeletion syndrome | 199318 | 612001 | - | 1 |
| 15q24 microdeletion syndrome | 94065 | 613406 | - | 2 |
| 16p13.11 microdeletion syndrome | 261236 | - | - | 1 |
| 16p13.11 microduplication syndrome | 261243 | - | - | 4 |
| 17p11.2 microduplication syndrome | 1713 | 610883 | - | 9 |
| 17p13.3 microduplication syndrome | 217385 | 613215 | - | 1 |
| 17q12 microduplication syndrome | 261272 | 614526 | - | 2 |
| 17q21.31 microdeletion syndrome | 363958 | 610443 | - | 2 |
| 17q21.31 microduplication syndrome | 217340 | 613533 | - | 2 |
| 1p36 deletion syndrome | 1606 | 607872 | 616975 | 25 |
| 1q21.1 microdeletion syndrome | 250989 | 612474 | - | 1 |
| 1q21.1 microduplication syndrome | 250994 | 612475 | - | 2 |
| 22q11.2 deletion syndrome | 567 | 188400 | 192430 | 117 |
| 22q11.2 microduplication syndrome | 1727 | 608363 | - | 11 |
| 2p15p16.1 microdeletion syndrome | 261349 | 612513 | - | 1 |
| 2p21 microdeletion syndrome | 163693 | 606407 | - | 1 |
| 2q23.1 microdeletion syndrome | 228402 | 156200 | - | 1 |
| 2q31.1 microdeletion syndrome | 251014 | - | - | 1 |
| 3-methylglutaconic aciduria type 3 | 67047 | 258501 | - | 1 |
| 3C syndrome | 7 | 220210 | 300963 | 2 |
| 3M syndrome | 2616 | 273750 | 612921 | 1 |
| 3q29 microdeletion syndrome | 65286 | 609425 | - | 3 |
| 3q29 microduplication syndrome | 251038 | 611936 | - | 3 |
| 48,XXXY syndrome | 96263 | - | - | 2 |
| 48,XXYY syndrome | 10 | - | - | 6 |
| 4q21 microdeletion syndrome | 238750 | 613509 | - | 1 |
| 5p13 microduplication syndrome | 329802 | 613174 | - | 1 |
| 5q35 microduplication syndrome | 228415 | - | - | 1 |
| 7q11.23 microduplication syndrome | 96121 | 609757 | - | 2 |
| 8p23.1 duplication syndrome | 251076 | - | - | 3 |
| 8q22.1 microdeletion syndrome | 178303 | 608156 | - | 1 |
| 8q24.3 deletion syndrome | 508488 | 615583 | - | 1 |
| Aarskog-Scott syndrome | 915 | 100050 | 305400 | 4 |
| Abetalipoproteinemia | 14 | 200100 | - | 2 |
| Acalvaria | 945 | - | - | 1 |
| Achondrogenesis | 932 | 200600 | 200610 | 1 |
| Achondroplasia | 15 | 100800 | - | 21 |
| Achromatopsia | 49382 | 216900 | 262300 | 16 |
| Acrocallosal syndrome | 36 | 200990 | - | 2 |
| Acrodermatitis enteropathica | 37 | 201100 | - | 4 |
| Acrodysostosis | 950 | 101800 | 614613 | 4 |
| Acromesomelic dysplasia, Hunter-Thompson type | 968 | 201250 | - | 1 |
| Acromicric dysplasia | 969 | 102370 | - | 2 |
| Acroosteolysis dominant type | 955 | 102400 | 102500 | 3 |
| Acropectorovertebral dysplasia | 957 | 102510 | - | 1 |
| Actinic prurigo | 330061 | 174770 | - | 2 |
| Acute necrotizing encephalopathy of childhood | 263524 | 614212 | - | 2 |
| Adams-Oliver syndrome | 974 | 100300 | 614219 | 5 |
| Adenylosuccinate lyase deficiency | 46 | 103050 | - | 1 |
| ADNP syndrome | 404448 | 615873 | - | 2 |
| ADULT syndrome | 978 | 103285 | - | 1 |
| AHDC1-related intellectual disability-obstructive sleep apnea-mild dysmorphism syndrome | 412069 | 615829 | - | 2 |
| Aicardi syndrome | 50 | 304050 | - | 14 |
| Aicardi-Goutières syndrome | 51 | 114100 | 225750 | 5 |
| Allan-Herndon-Dudley syndrome | 59 | 300523 | - | 1 |
| Allergic bronchopulmonary aspergillosis | 1164 | 103920 | - | 1 |
| Alobar holoprosencephaly | 93925 | 157170 | 609637 | 1 |
| Alpha-mannosidosis | 61 | 248500 | - | 1 |
| Alpha-thalassemia-X-linked intellectual disability syndrome | 847 | 301040 | - | 1 |
| Alport syndrome | 63 | 104200 | 203780 | 17 |
| Alternating hemiplegia of childhood | 2131 | 104290 | 614820 | 9 |
| Amelogenesis imperfecta | 88661 | 104500 | 104510 | 4 |
| Anaphylactoid purpura | 761 | - | - | 48 |
| Andersen-Tawil syndrome | 37553 | 170390 | - | 2 |
| Androgen insensitivity syndrome | 754 | 0 | - | 2 |
| Angelman syndrome | 72 | 105830 | - | 76 |
| Angelman syndrome due to imprinting defect in 15q11-q13 | 411515 | - | - | 2 |
| Angioosteohypertrophic syndrome | 2346 | 149000 | 608355 | 3 |
| Aniridia-cerebellar ataxia-intellectual disability syndrome | 1065 | 206700 | - | 2 |
| Ankyloblepharon-ectodermal defects-cleft lip/palate syndrome | 1071 | 106260 | - | 1 |
| Annular pancreas | 675 | 167750 | - | 1 |
| Anotia | 93976 | 600674 | - | 1 |
| Aorta coarctation | 1457 | 120000 | - | 5 |
| Aortic arch interruption | 2299 | - | - | 5 |
| Apert syndrome | 87 | 101200 | - | 45 |
| Aplasia cutis congenita | 1114 | 107600 | - | 3 |
| Arachnoid cyst | 2356 | 182990 | 207790 | 24 |
| Argininemia | 90 | 207800 | - | 3 |
| Arterial tortuosity syndrome | 3342 | 208050 | - | 1 |
| Arthrogryposis multiplex congenita | 1037 | - | - | 13 |
| Ataxia-hypogonadism-choroidal dystrophy syndrome | 1180 | 215470 | - | 2 |
| Ataxia-intellectual disability-oculomotor apraxia-cerebellar cysts syndrome | 370022 | 615960 | - | 1 |
| Ataxia-telangiectasia | 100 | 208900 | 208910 | 16 |
| Atelosteogenesis type II | 56304 | 256050 | - | 1 |
| Atelosteogenesis type III | 56305 | 108721 | - | 1 |
| Atresia of small intestine | 1201 | 243600 | - | 1 |
| Atypical coarctation of aorta | 1456 | - | - | 1 |
| Atypical Rett syndrome | 3095 | 300672 | 312750 | 1 |
| Atypical teratoid rhabdoid tumor | 99966 | 609322 | - | 22 |
| Autism spectrum disorder due to AUTS2 deficiency | 352490 | 615834 | - | 2 |
| Autoimmune polyendocrinopathy type 1 | 3453 | 240300 | - | 2 |
| Autosomal dominant Charcot-Marie-Tooth disease type 2A2 | 99947 | 609260 | - | 1 |
| Autosomal dominant epidermolytic ichthyosis | 312 | 113800 | 607602 | 2 |
| Autosomal dominant hyper-IgE syndrome | 2314 | 147060 | - | 5 |
| Autosomal dominant limb-girdle muscular dystrophy type 1B | 264 | 159001 | 181350 | 1 |
| Autosomal dominant nocturnal frontal lobe epilepsy | 98784 | 600513 | 603204 | 2 |
| Autosomal dominant optic atrophy | 98672 | - | - | 1 |
| Autosomal dominant optic atrophy, classic form | 98673 | 165500 | 605293 | 1 |
| Autosomal erythropoietic protoporphyria | 79278 | 177000 | - | 5 |
| Autosomal recessive centronuclear myopathy | 169186 | 255200 | 615959 | 1 |
| Autosomal recessive dopa-responsive dystonia | 101150 | 605407 | - | 2 |
| Autosomal recessive limb-girdle muscular dystrophy type 2C | 353 | 253700 | - | 1 |
| Autosomal recessive limb-girdle muscular dystrophy type 2D | 62 | 608099 | - | 4 |
| Autosomal recessive limb-girdle muscular dystrophy type 2E | 119 | 604286 | - | 1 |
| Autosomal recessive limb-girdle muscular dystrophy type 2S | 369840 | 615356 | - | 1 |
| Autosomal recessive limb-girdle muscular dystrophy type 2T | 363623 | 615352 | - | 1 |
| Autosomal recessive multiple pterygium syndrome | 2990 | 265000 | - | 5 |
| Autosomal recessive polycystic kidney disease | 731 | 263200 | 617610 | 5 |
| Autosomal recessive sideroblastic anemia | 260305 | 182170 | 205950 | 1 |
| Autosomal recessive spastic paraplegia type 56 | 320411 | 615030 | - | 1 |
| Autosomal recessive spondylocostal dysostosis | 2311 | 277300 | 608681 | 1 |
| Axenfeld-Rieger syndrome | 782 | 180500 | 601499 | 5 |
| Aymé-Gripp syndrome | 1272 | 601088 | 601353 | 2 |
| Baller-Gerold syndrome | 1225 | 218600 | - | 1 |
| Bannayan-Riley-Ruvalcaba syndrome | 109 | 158350 | - | 3 |
| Bardet-Biedl syndrome | 110 | 209900 | 600151 | 10 |
| Barth syndrome | 111 | 302060 | - | 6 |
| Becker muscular dystrophy | 98895 | 159050 | 300376 | 11 |
| Beckwith-Wiedemann syndrome | 116 | 130650 | - | 19 |
| Benign childhood occipital epilepsy, Panayiotopoulos type | 98815 | - | - | 3 |
| Benign hereditary chorea | 1429 | 118700 | 215450 | 1 |
| Benign paroxysmal torticollis of infancy | 71518 | - | - | 2 |
| Berardinelli-Seip congenital lipodystrophy | 528 | 269700 | 608594 | 1 |
| Best vitelliform macular dystrophy | 1243 | 153700 | - | 2 |
| Beta-ketothiolase deficiency | 134 | 203750 | - | 1 |
| Beta-propeller protein-associated neurodegeneration | 329284 | 300894 | - | 1 |
| Beta-thalassemia | 848 | 603902 | 613985 | 13 |
| Beta-thalassemia major | 231214 | 613985 | - | 6 |
| Bethlem myopathy | 610 | 158810 | 616471 | 5 |
| Bilirubin encephalopathy | 415286 | - | - | 10 |
| Biotinidase deficiency | 79241 | 253260 | - | 2 |
| Blackfan-Diamond anemia | 124 | 105650 | 300946 | 18 |
| Bladder exstrophy | 93930 | 600057 | - | 13 |
| Blau syndrome | 90340 | - | - | 1 |
| Blepharophimosis-epicanthus inversus-ptosis syndrome | 126 | 110100 | - | 6 |
| Blepharophimosis-intellectual disability syndrome, Ohdo type | 2728 | 249620 | - | 5 |
| Bloom syndrome | 125 | 210900 | - | 4 |
| Blount disease | 2768 | 188700 | 259200 | 12 |
| Blue rubber bleb nevus | 1059 | 112200 | - | 1 |
| Bohring-Opitz syndrome | 97297 | 605039 | - | 8 |
| Borjeson-Forssman-Lehmann syndrome | 127 | 301900 | - | 3 |
| Brachydactyly type C | 93384 | 113100 | - | 1 |
| Brachytelephalangic chondrodysplasia punctata | 79345 | 302950 | 602497 | 1 |
| Brain-lung-thyroid syndrome | 209905 | 610978 | - | 2 |
| Branchio-oculo-facial syndrome | 1297 | 113620 | - | 1 |
| Bronchopulmonary dysplasia | 70589 | - | - | 2 |
| Bruck syndrome | 2771 | 259450 | 609220 | 1 |
| C syndrome | 1308 | 211750 | - | 1 |
| Campomelic dysplasia | 140 | 114290 | 211990 | 4 |
| Camptodactyly-arthropathy-coxa-vara-pericarditis syndrome | 2848 | 208250 | - | 1 |
| Camptodactyly-tall stature-scoliosis-hearing loss syndrome | 85164 | 610474 | - | 1 |
| Canavan disease | 141 | 271900 | - | 2 |
| Capillary malformation-arteriovenous malformation | 137667 | 608354 | - | 1 |
| Cardiofaciocutaneous syndrome | 1340 | 115150 | 615278 | 9 |
| Carney complex | 1359 | 160980 | 605244 | 7 |
| Carnitine palmitoyl transferase 1A deficiency | 156 | 255120 | - | 1 |
| Carnitine-acylcarnitine translocase deficiency | 159 | 212138 | - | 1 |
| Carpenter syndrome | 65759 | 201000 | 614976 | 1 |
| Cartilage-hair hypoplasia | 175 | 250250 | 250460 | 1 |
| Cat-eye syndrome | 195 | 115470 | - | 3 |
| Catecholaminergic polymorphic ventricular tachycardia | 3286 | 604772 | 611938 | 1 |
| Catel-Manzke syndrome | 1388 | 302380 | 616145 | 2 |
| Caudal regression sequence | 3027 | 600145 | - | 11 |
| CEDNIK syndrome | 66631 | 609528 | - | 1 |
| Central core disease | 597 | 117000 | - | 1 |
| Central diabetes insipidus | 178029 | 125700 | 304900 | 1 |
| Central nervous system primitive neuroectodermal tumor | 251870 | - | - | 1 |
| Central precocious puberty | 759 | 176400 | 615346 | 2 |
| Centronuclear myopathy | 595 | - | - | 6 |
| Cerebellar-facial-dental syndrome | 444072 | 616202 | - | 1 |
| Cerebral visual impairment | 447788 | - | - | 9 |
| Cerebrofacioarticular syndrome | 314679 | 601390 | 615546 | 1 |
| Cerebrotendinous xanthomatosis | 909 | 213700 | - | 3 |
| CHARGE syndrome | 138 | 214800 | - | 58 |
| Chédiak-Higashi syndrome | 167 | 214500 | - | 5 |
| Cherubism | 184 | 118400 | - | 4 |
| Childhood absence epilepsy | 64280 | 600131 | 607681 | 2 |
| Childhood apraxia of speech | 209908 | 602081 | - | 35 |
| Childhood disintegrative disorder | 168782 | - | - | 2 |
| CHIME syndrome | 3474 | 280000 | - | 1 |
| Choanal atresia | 137914 | 608911 | - | 3 |
| Choanal atresia-hearing loss-cardiac defects-craniofacial dysmorphism syndrome | 1200 | 608572 | 616462 | 1 |
| Choledochal cyst | 480501 | - | - | 2 |
| Choroid plexus carcinoma | 251899 | 260500 | - | 2 |
| Christianson syndrome | 85278 | 300243 | - | 5 |
| Chronic nonbacterial osteomyelitis/Chronic recurrent multifocal osteomyelitis | 324964 | 259680 | - | 2 |
| CINCA syndrome | 1451 | 607115 | - | 1 |
| Classic galactosemia | 79239 | 230400 | - | 2 |
| Classic homocystinuria | 394 | 236200 | - | 1 |
| Classic phenylketonuria | 79254 | - | - | 2 |
| Cleft lip/palate | 199306 | 119530 | 129400 | 93 |
| Cleft palate | 2014 | 119540 | - | 16 |
| Cleft velum | 99772 | 119570 | - | 1 |
| Cleidocranial dysplasia | 1452 | 119600 | 216330 | 5 |
| CLN3 disease | 228346 | 204200 | - | 1 |
| CLN7 disease | 228366 | 610951 | - | 2 |
| Cloacal exstrophy | 93929 | 258040 | - | 7 |
| CLOVES syndrome | 140944 | 612918 | - | 3 |
| Coats disease | 190 | 300216 | - | 14 |
| Coffin-Lowry syndrome | 192 | 303600 | - | 4 |
| Coffin-Siris syndrome | 1465 | 135900 | 614607 | 19 |
| Cohen syndrome | 193 | 216550 | - | 7 |
| Cone rod dystrophy | 1872 | 120970 | 300476 | 2 |
| Congenital amegakaryocytic thrombocytopenia | 3319 | 604498 | - | 1 |
| Congenital and infantile nephrotic syndrome | 97556 | - | - | 5 |
| Congenital cataract-hypertrophic cardiomyopathy-mitochondrial myopathy syndrome | 1369 | 212350 | 615418 | 1 |
| Congenital chylothorax | 264688 | 603523 | - | 2 |
| Congenital contractural arachnodactyly | 115 | 121050 | - | 3 |
| Congenital deformities of limbs | 294944 | - | - | 1 |
| Congenital diaphragmatic hernia | 2140 | 142340 | 222400 | 35 |
| Congenital disorder of glycosylation | 137 | - | - | 3 |
| Congenital dyserythropoietic anemia | 85 | - | - | 2 |
| Congenital dyserythropoietic anemia type II | 98873 | 224100 | - | 1 |
| Congenital fiber-type disproportion myopathy | 2020 | 255310 | 300580 | 1 |
| Congenital fibrosis of extraocular muscles | 45358 | 135700 | 600638 | 1 |
| Congenital glaucoma | 98976 | 231300 | 600975 | 12 |
| Congenital heart block | 60041 | 234700 | - | 2 |
| Congenital hydrocephalus | 2185 | 236600 | 615219 | 4 |
| Congenital hypothyroidism | 442 | - | - | 19 |
| Congenital insensitivity to pain with hyperhidrosis | 217399 | - | - | 2 |
| Congenital lactase deficiency | 53690 | 223000 | - | 1 |
| Congenital microcephaly-severe encephalopathy-progressive cerebral atrophy syndrome | 391376 | 615574 | - | 1 |
| Congenital muscular dystrophy | 97242 | - | - | 1 |
| Congenital muscular dystrophy due to LMNA mutation | 157973 | 613205 | - | 1 |
| Congenital muscular dystrophy type 1A | 258 | 607855 | 618138 | 2 |
| Congenital muscular dystrophy, Ullrich type | 75840 | 254090 | 616470 | 2 |
| Congenital myasthenic syndrome | 590 | 254190 | 254210 | 4 |
| Congenital myopathy | 97245 | - | - | 2 |
| Congenital nephrotic syndrome, Finnish type | 839 | 256300 | - | 1 |
| Congenital pseudoarthrosis of the clavicle | 66630 | 118980 | - | 1 |
| Congenital pseudoarthrosis of the tibia | 295018 | - | - | 2 |
| Congenital ptosis | 91411 | 178300 | 300245 | 4 |
| Congenital reticular ichthyosiform erythroderma | 281190 | 609165 | - | 1 |
| Congenital rubella syndrome | 290 | - | - | 3 |
| Congenital stationary night blindness | 215 | 163500 | 257270 | 2 |
| Congenital sucrase-isomaltase deficiency | 35122 | 222900 | - | 18 |
| Congenital toxoplasmosis | 858 | - | - | 2 |
| Congenital tracheal stenosis | 141127 | 603569 | - | 2 |
| Congenital vertical talus | 178382 | 192950 | - | 2 |
| Constitutional mismatch repair deficiency syndrome | 252202 | 276300 | - | 1 |
| Continuous spikes and waves during sleep | 725 | 245570 | - | 4 |
| Cornelia de Lange syndrome | 199 | 122470 | 300590 | 16 |
| Corpus callosum agenesis-neuronopathy syndrome | 1496 | 218000 | - | 1 |
| Costello syndrome | 3071 | 218040 | - | 7 |
| Cranioectodermal dysplasia | 1515 | 218330 | 613610 | 1 |
| Craniofrontonasal dysplasia | 1520 | 304110 | - | 1 |
| Craniosynostosis | 1531 | - | - | 92 |
| Crigler-Najjar syndrome | 205 | 218800 | 606785 | 2 |
| Criss-cross heart | 1461 | - | - | 2 |
| Crouzon disease | 207 | 123500 | - | 1 |
| Cryopyrin-associated periodic syndrome | 208650 | - | - | 1 |
| Cutaneous mastocytosis | 66646 | - | - | 1 |
| Cutis marmorata telangiectatica congenita | 1556 | 219250 | - | 6 |
| Cystic hygroma | 79486 | 257350 | - | 8 |
| De Barsy syndrome | 2962 | 219150 | 614438 | 1 |
| Deafness-infertility syndrome | 94064 | 611102 | - | 2 |
| Dehydrated hereditary stomatocytosis | 3202 | 194380 | 616689 | 2 |
| Dense deposit disease | 93571 | 609814 | - | 2 |
| Dent disease | 1652 | 300009 | 300554 | 2 |
| Dentinogenesis imperfecta | 49042 | - | - | 3 |
| Denys-Drash syndrome | 220 | 194080 | - | 1 |
| Diastrophic dwarfism | 628 | 222600 | - | 3 |
| Diencephalic syndrome | 1672 | - | - | 1 |
| Distal 16p11.2 microdeletion syndrome | 261222 | 613444 | - | 1 |
| Distal 7q11.23 microduplication syndrome | 261102 | - | - | 2 |
| Distal monosomy 15q | 1596 | 612626 | - | 1 |
| Distal monosomy 3p | 1620 | 613792 | - | 1 |
| Distal tetrasomy 15q | 314588 | 614846 | - | 1 |
| Distal trisomy 10q | 96102 | - | - | 1 |
| DOORS syndrome | 79500 | 220500 | - | 1 |
| Dopa-responsive dystonia | 255 | - | - | 2 |
| Dopamine beta-hydroxylase deficiency | 230 | 223360 | - | 1 |
| Double outlet right ventricle | 3426 | 217095 | - | 7 |
| Down syndrome | 870 | 190685 | - | 145 |
| Dravet syndrome | 33069 | 607208 | 612164 | 46 |
| Duane retraction syndrome | 233 | 126800 | 604356 | 17 |
| Dubowitz syndrome | 235 | 223370 | - | 2 |
| Duchenne and Becker muscular dystrophy | 262 | - | - | 15 |
| Duchenne muscular dystrophy | 98896 | 310200 | - | 87 |
| Duodenal atresia | 1203 | 223400 | - | 2 |
| Dyskeratosis congenita | 1775 | 127550 | 224230 | 3 |
| Dysplasia epiphysealis hemimelica | 1822 | 127800 | - | 1 |
| Dystrophic epidermolysis bullosa | 303 | - | - | 1 |
| Early infantile epileptic encephalopathy | 1934 | 300672 | 308350 | 5 |
| Early myoclonic encephalopathy | 1935 | 609304 | 616341 | 1 |
| Early-onset schizophrenia | 96369 | - | - | 1 |
| Ectodermal dysplasia syndrome | 79373 | - | - | 1 |
| EEC syndrome | 1896 | 129900 | 604292 | 2 |
| Ehlers-Danlos syndrome | 98249 | - | - | 93 |
| Ehlers-Danlos syndrome, classic type | 287 | 130000 | - | 1 |
| Ehlers-Danlos syndrome, kyphoscoliotic type | 1900 | 225400 | - | 1 |
| Ehlers-Danlos syndrome, periodontitis type | 75392 | 130080 | 617174 | 1 |
| Ehlers-Danlos syndrome, vascular type | 286 | 130050 | - | 2 |
| Ehlers-Danlos/osteogenesis imperfecta syndrome | 230857 | - | - | 1 |
| Ellis Van Creveld syndrome | 289 | 225500 | 617088 | 1 |
| Emanuel syndrome | 96170 | 609029 | - | 6 |
| Emery-Dreifuss muscular dystrophy | 261 | 181350 | 300696 | 9 |
| Encephalopathy due to GLUT1 deficiency | 71277 | 606777 | - | 8 |
| Enthesitis-related juvenile idiopathic arthritis | 85438 | - | - | 2 |
| Ependymoblastoma | 251880 | - | - | 3 |
| Epidermal nevus syndrome | 35125 | - | - | 1 |
| Epidermolysis bullosa simplex | 304 | - | - | 2 |
| Epidermolysis bullosa simplex, generalized severe | 79396 | 131760 | - | 1 |
| Epiphysiolysis of the hip | 399329 | - | - | 4 |
| Epithelial recurrent erosion dystrophy | 293381 | 122400 | - | 1 |
| Erythrokeratodermia variabilis | 317 | 133200 | 617524 | 2 |
| Esophageal atresia | 1199 | 189960 | - | 23 |
| Ewing sarcoma | 319 | 612219 | - | 54 |
| Exstrophy-epispadias complex | 322 | 258040 | 600057 | 2 |
| Fabry disease | 324 | 301500 | - | 11 |
| Familial afibrinogenemia | 98880 | 202400 | - | 1 |
| Familial cold urticaria | 47045 | 120100 | 616115 | 2 |
| Familial dysautonomia | 1764 | 223900 | - | 8 |
| Familial exudative vitreoretinopathy | 891 | 133780 | 305390 | 1 |
| Familial hemophagocytic lymphohistiocytosis | 540 | 267700 | 603552 | 1 |
| Familial long QT syndrome | 768 | 192500 | 220400 | 1 |
| Familial paroxysmal ataxia | 97 | 108500 | - | 2 |
| Fanconi anemia | 84 | 227645 | 227646 | 23 |
| Farber disease | 333 | 228000 | - | 1 |
| FATCO syndrome | 2492 | - | - | 1 |
| Feingold syndrome | 1305 | 164280 | 614326 | 2 |
| Feingold syndrome type 1 | 391641 | 164280 | - | 1 |
| Femoral-facial syndrome | 1988 | 134780 | - | 2 |
| Femur-fibula-ulna complex | 2019 | 228200 | - | 24 |
| Fetal alcohol syndrome | 1915 | - | - | 59 |
| Fetal valproate syndrome | 1906 | 609442 | - | 4 |
| Fibrodysplasia ossificans progressiva | 337 | 135100 | - | 4 |
| Fibular hemimelia | 93323 | - | - | 24 |
| Focal dermal hypoplasia | 2092 | 305600 | - | 3 |
| Focal facial dermal dysplasia type III | 1807 | 227260 | - | 1 |
| Fowler syndrome | 221126 | 225790 | - | 11 |
| Fragile X syndrome | 908 | 300624 | - | 32 |
| Fraser syndrome | 2052 | 219000 | 617666 | 3 |
| Freeman-Sheldon syndrome | 2053 | 193700 | 277720 | 6 |
| Friedreich ataxia | 95 | 229300 | 601992 | 56 |
| Frontometaphyseal dysplasia | 1826 | 305620 | 617137 | 1 |
| Fucosidosis | 349 | 230000 | - | 1 |
| Fumaric aciduria | 24 | 606812 | - | 1 |
| Gabriele-de Vries syndrome | 506358 | 617557 | - | 1 |
| Galactosemia | 352 | 230200 | 230350 | 70 |
| Gastroschisis | 2368 | 230750 | - | 95 |
| Gaucher disease type 2 | 77260 | 230900 | - | 2 |
| Geleophysic dysplasia | 2623 | 231050 | 614185 | 2 |
| Generalized dominant dystrophic epidermolysis bullosa | 231568 | 131750 | - | 1 |
| Generalized epilepsy with febrile seizures-plus | 36387 | 604233 | 604403 | 2 |
| Generalized resistance to thyroid hormone | 3221 | 188570 | 274300 | 2 |
| Genitopatellar syndrome | 85201 | 606170 | - | 1 |
| Geroderma osteodysplastica | 2078 | 231070 | - | 1 |
| Giant axonal neuropathy | 643 | 256850 | - | 1 |
| Gitelman syndrome | 358 | 263800 | - | 12 |
| Glanzmann thrombasthenia | 849 | 273800 | - | 2 |
| Glucose-galactose malabsorption | 35710 | 606824 | - | 2 |
| Glutaryl-CoA dehydrogenase deficiency | 25 | 231670 | - | 5 |
| Glycogen storage disease due to glucose-6-phosphatase deficiency | 364 | 232200 | 232220 | 1 |
| Glycogen storage disease due to glucose-6-phosphatase deficiency type Ia | 79258 | 232200 | - | 1 |
| Glycogen storage disease due to glycogen debranching enzyme deficiency | 366 | 232400 | - | 1 |
| Glycogen storage disease due to LAMP-2 deficiency | 34587 | 300257 | - | 4 |
| Glycogen storage disease due to liver glycogen phosphorylase deficiency | 369 | 232700 | - | 1 |
| Glycogen storage disease due to muscle glycogen phosphorylase deficiency | 368 | 232600 | - | 2 |
| GM1 gangliosidosis | 354 | 230500 | 230600 | 3 |
| Goldberg-Shprintzen megacolon syndrome | 66629 | 609460 | - | 2 |
| Goldenhar syndrome | 374 | 164210 | - | 31 |
| Gómez-López-Hernández syndrome | 1532 | 601853 | - | 1 |
| Granular corneal dystrophy type II | 98963 | 607541 | - | 1 |
| Greig cephalopolysyndactyly syndrome | 380 | 175700 | - | 1 |
| Griscelli syndrome | 381 | 214450 | 607624 | 1 |
| Haddad syndrome | 99803 | 209880 | - | 1 |
| Hallermann-Streiff syndrome | 2108 | 234100 | - | 1 |
| Harlequin ichthyosis | 457 | 242500 | - | 4 |
| Heiner syndrome | 99932 | - | - | 1 |
| Hemimegalencephaly | 99802 | - | - | 4 |
| Hemolytic anemia due to red cell pyruvate kinase deficiency | 766 | 266200 | - | 4 |
| Hemophagocytic syndrome | 158032 | - | - | 9 |
| Hemophilia | 448 | - | - | 87 |
| Hemophilia A | 98878 | 306700 | - | 1 |
| Hemophilia B | 98879 | 306900 | - | 8 |
| Hennekam syndrome | 2136 | 235510 | 616006 | 2 |
| Hepatoblastoma | 449 | 114550 | - | 10 |
| Hereditary chronic pancreatitis | 676 | 167800 | - | 4 |
| Hereditary hyperekplexia | 3197 | 149400 | 614618 | 7 |
| Hereditary methemoglobinemia | 621 | 250700 | 250790 | 1 |
| Hereditary retinoblastoma | 357027 | 180200 | - | 1 |
| Hermansky-Pudlak syndrome | 79430 | 203300 | 608233 | 4 |
| Heterotaxia | 450 | 270100 | 306955 | 5 |
| Hidrotic ectodermal dysplasia | 189 | 129500 | - | 2 |
| Hirschsprung disease | 388 | 142623 | 600155 | 62 |
| Holoprosencephaly | 2162 | 142945 | 142946 | 22 |
| Holt-Oram syndrome | 392 | 142900 | - | 7 |
| Homocystinuria due to methylene tetrahydrofolate reductase deficiency | 395 | 236250 | - | 2 |
| Homozygous familial hypercholesterolemia | 391665 | 143890 | 602247 | 2 |
| Hurler syndrome | 93473 | 607014 | - | 9 |
| Hutchinson-Gilford progeria syndrome | 740 | 176670 | - | 69 |
| Hydranencephaly | 2177 | - | - | 12 |
| Hydroa vacciniforme | 330058 | - | - | 2 |
| Hydrops fetalis | 1041 | 236750 | - | 10 |
| Hyperimmunoglobulinemia D with periodic fever | 343 | 260920 | - | 1 |
| Hyperkalemic periodic paralysis | 682 | 170500 | - | 3 |
| Hyperphosphatasia-intellectual disability syndrome | 247262 | 239300 | 614207 | 1 |
| Hypertrichotic osteochondrodysplasia, Cantu type | 1517 | 239850 | - | 2 |
| Hypochondroplasia | 429 | 146000 | - | 3 |
| Hypodontia-dysplasia of nails syndrome | 2228 | 189500 | - | 1 |
| Hypoglossia-hypodactyly syndrome | 989 | 103300 | - | 2 |
| Hypohidrotic ectodermal dysplasia | 238468 | 129490 | 224900 | 1 |
| Hypokalemic periodic paralysis | 681 | 170400 | 613345 | 8 |
| Hypomyelination with atrophy of basal ganglia and cerebellum | 139441 | 612438 | 617899 | 2 |
| Hypoplastic left heart syndrome | 2248 | 241550 | 614435 | 117 |
| Hypoplastic right heart syndrome | 98723 | - | - | 4 |
| Hypotonia-speech impairment-severe cognitive delay syndrome | 371364 | 615419 | 616801 | 1 |
| Idiopathic juvenile osteoporosis | 85193 | 259750 | 615221 | 2 |
| IMAGe syndrome | 85173 | 614732 | - | 1 |
| Incontinentia pigmenti | 464 | 308300 | - | 14 |
| Infant botulism | 178478 | - | - | 2 |
| Infantile cerebellar-retinal degeneration | 313850 | 614559 | - | 1 |
| Infantile hypophosphatasia | 247651 | 241500 | - | 2 |
| Infantile myofibromatosis | 2591 | 228550 | 615293 | 2 |
| Infantile neuroaxonal dystrophy | 35069 | 256600 | 610217 | 1 |
| Infantile systemic hyalinosis | 2176 | 228600 | - | 1 |
| Infantile-onset ascending hereditary spastic paralysis | 293168 | 607225 | - | 1 |
| Inherited congenital spastic tetraplegia | 210141 | 603513 | 612900 | 2 |
| Iniencephaly | 63259 | - | - | 1 |
| Intellectual disability-cataracts-calcified pinnae-myopathy syndrome | 3042 | 259050 | - | 2 |
| Intellectual disability-developmental delay-contractures syndrome | 3454 | 314580 | - | 2 |
| Intellectual disability-seizures-abnormal gait-facial dysmorphism syndrome | 513456 | 617616 | - | 1 |
| Intellectual disability-seizures-hypophosphatasia-ophthalmic-skeletal anomalies syndrome | 369837 | 615398 | - | 1 |
| Intellectual disability-sparse hair-brachydactyly syndrome | 3051 | 601358 | - | 1 |
| Interatrial communication | 1478 | 108800 | 607941 | 8 |
| Intermediate uveitis | 279914 | - | - | 1 |
| Isolated split hand-split foot malformation | 2440 | 183600 | 225300 | 5 |
| Isovaleric acidemia | 33 | 243500 | - | 4 |
| Ito hypomelanosis | 435 | 300337 | - | 2 |
| Ivemark syndrome | 97548 | 208530 | - | 3 |
| Jacobsen syndrome | 2308 | 147791 | - | 8 |
| Jeavons syndrome | 139431 | - | - | 5 |
| Jervell and Lange-Nielsen syndrome | 90647 | 220400 | 612347 | 1 |
| Jeune syndrome | 474 | 208500 | 611263 | 3 |
| Johanson-Blizzard syndrome | 2315 | 243800 | - | 2 |
| Joubert syndrome | 475 | 213300 | 610688 | 26 |
| Joubert syndrome and related disorders | 140874 | - | - | 2 |
| Juvenile dermatomyositis | 93672 | - | - | 21 |
| Juvenile Huntington disease | 248111 | 143100 | - | 12 |
| Juvenile hyaline fibromatosis | 2028 | 228600 | - | 1 |
| Juvenile idiopathic arthritis | 92 | - | - | 47 |
| Juvenile myelomonocytic leukemia | 86834 | 607785 | - | 14 |
| Juvenile myoclonic epilepsy | 307 | 254770 | 604827 | 6 |
| Juvenile neuronal ceroid lipofuscinosis | 79264 | 204200 | 204500 | 56 |
| Juvenile xanthogranuloma | 158000 | - | - | 2 |
| Kabuki syndrome | 2322 | 147920 | 300867 | 29 |
| Kallmann syndrome | 478 | 147950 | 244200 | 8 |
| KBG syndrome | 2332 | 148050 | - | 5 |
| Kenny-Caffey syndrome | 2333 | 127000 | 244460 | 3 |
| Keratosis follicularis spinulosa decalvans | 2340 | 308800 | 604093 | 2 |
| KID syndrome | 477 | 148210 | 242150 | 1 |
| Kleefstra syndrome | 261494 | 610253 | - | 28 |
| Klippel-Trénaunay syndrome | 90308 | 149000 | - | 14 |
| Kniest dysplasia | 485 | 156550 | - | 1 |
| Koolen-De Vries syndrome | 96169 | 610443 | - | 9 |
| L1 syndrome | 275543 | 303350 | 304100 | 2 |
| Lacrimoauriculodentodigital syndrome | 2363 | 149730 | - | 2 |
| Lafora disease | 501 | 254780 | - | 1 |
| Lamellar ichthyosis | 313 | 242300 | 601277 | 3 |
| Landau-Kleffner syndrome | 98818 | 245570 | - | 5 |
| Langer mesomelic dysplasia | 2632 | 249700 | - | 1 |
| Large congenital melanocytic nevus | 626 | 137550 | - | 2 |
| Laron syndrome | 633 | 262500 | - | 4 |
| Larsen syndrome | 503 | 150250 | - | 4 |
| Laryngotracheoesophageal cleft | 2004 | 215800 | - | 1 |
| Late infantile neuronal ceroid lipofuscinosis | 168491 | 204500 | 256730 | 1 |
| Lateral meningocele syndrome | 2789 | 130720 | - | 2 |
| Laurence-Moon syndrome | 2377 | 245800 | - | 1 |
| Leber congenital amaurosis | 65 | 179900 | 204000 | 7 |
| Legg-Calvé-Perthes disease | 2380 | 150600 | - | 12 |
| Legius syndrome | 137605 | 611431 | - | 2 |
| Lemierre syndrome | 137839 | - | - | 4 |
| Lennox-Gastaut syndrome | 2382 | 615369 | 616346 | 12 |
| Leprechaunism | 508 | 246200 | - | 2 |
| Léri-Weill dyschondrosteosis | 240 | 127300 | - | 2 |
| Lesch-Nyhan syndrome | 510 | 300322 | 308950 | 7 |
| Lethal ataxia with deafness and optic atrophy | 1187 | 301835 | - | 1 |
| Leukoencephalopathy with brain stem and spinal cord involvement-high lactate syndrome | 137898 | 611105 | - | 2 |
| Leukomelanoderma-infantilism-intellectual disability-hypodontia-hypotrichosis syndrome | 1816 | 246500 | - | 1 |
| Liddle syndrome | 526 | 177200 | 618114 | 1 |
| Ligneous conjunctivitis | 97231 | 217090 | - | 1 |
| Limb body wall complex | 2369 | - | - | 6 |
| Linear nevus sebaceus syndrome | 2612 | 163200 | - | 1 |
| Lissencephaly | 48471 | - | - | 43 |
| Lobar holoprosencephaly | 93924 | 157170 | 609637 | 1 |
| Loeys-Dietz syndrome | 60030 | 609192 | 610168 | 6 |
| Loose anagen syndrome | 168 | 600628 | - | 3 |
| LUMBAR syndrome | 83628 | - | - | 2 |
| Lysinuric protein intolerance | 470 | 222700 | - | 1 |
| Macrocephaly-intellectual disability-neurodevelopmental disorder-small thorax syndrome | 457485 | 616638 | - | 1 |
| Macrothrombocytopenia-lymphedema-developmental delay-facial dysmorphism-camptodactyly syndrome | 487796 | 616737 | - | 1 |
| Madelung deformity | 35688 | 127300 | - | 7 |
| Maffucci syndrome | 163634 | 614569 | - | 1 |
| Mal de Meleda | 87503 | 248300 | - | 2 |
| Malan overgrowth syndrome | 420179 | 614753 | - | 2 |
| Malignant migrating partial seizures of infancy | 293181 | 613722 | 614959 | 1 |
| Mandibular hypoplasia-deafness-progeroid features-lipodystrophy syndrome | 363649 | 615381 | - | 1 |
| Maple syrup urine disease | 511 | 248600 | 615135 | 29 |
| Marcus-Gunn syndrome | 91412 | 154600 | - | 10 |
| Marden-Walker syndrome | 2461 | 248700 | - | 1 |
| Marinesco-Sjögren syndrome | 559 | 248800 | - | 1 |
| Marshall syndrome | 560 | 154780 | - | 2 |
| Marshall-Smith syndrome | 561 | 602535 | - | 2 |
| Maternal phenylketonuria | 2209 | 261600 | - | 4 |
| Mayer-Rokitansky-Küster-Hauser syndrome | 3109 | 277000 | 601076 | 13 |
| McCune-Albright syndrome | 562 | 174800 | - | 7 |
| Meckel syndrome | 564 | 249000 | 603194 | 2 |
| Meconium aspiration syndrome | 70588 | - | - | 4 |
| Medium chain acyl-CoA dehydrogenase deficiency | 42 | 201450 | - | 5 |
| Megacystis-microcolon-intestinal hypoperistalsis syndrome | 2241 | 249210 | - | 3 |
| Megalencephaly-capillary malformation-polymicrogyria syndrome | 60040 | 602501 | - | 1 |
| Megalencephaly-polymicrogyria-postaxial polydactyly-hydrocephalus syndrome | 83473 | 603387 | 615937 | 1 |
| MEGDEL syndrome | 352328 | 614739 | - | 1 |
| Melkersson-Rosenthal syndrome | 2483 | 155900 | - | 2 |
| Menkes disease | 565 | 309400 | - | 2 |
| Metachondromatosis | 2499 | 156250 | - | 1 |
| Metatropic dysplasia | 2635 | 156530 | - | 1 |
| Micro syndrome | 2510 | 600118 | 614222 | 1 |
| Microphthalmia with linear skin defects syndrome | 2556 | 300887 | 300952 | 1 |
| Microphthalmia, Lenz type | 568 | 300166 | 309800 | 1 |
| Microtia | 83463 | 128800 | 600674 | 90 |
| Miller-Dieker syndrome | 531 | 247200 | - | 4 |
| Milroy disease | 79452 | 153100 | 247440 | 1 |
| MIRAGE syndrome | 494433 | 617053 | - | 2 |
| Moebius syndrome | 570 | 157900 | - | 26 |
| Monilethrix | 573 | 158000 | - | 6 |
| Monosomy 18q | 1600 | 601808 | - | 4 |
| Monosomy 21 | 574 | - | - | 2 |
| Monosomy 22q13 | 48652 | 606232 | - | 39 |
| Monosomy 5p | 281 | 123450 | - | 33 |
| Monosomy 9p | 261112 | 158170 | - | 3 |
| Morning glory disc anomaly | 35737 | 120430 | - | 3 |
| Mosaic trisomy 14 | 1703 | - | - | 2 |
| Mosaic trisomy 16 | 1708 | - | - | 1 |
| Mosaic trisomy 20 | 1724 | - | - | 1 |
| Mosaic trisomy 22 | 96068 | - | - | 1 |
| Mosaic trisomy 7 | 1747 | - | - | 1 |
| Mosaic trisomy 8 | 96061 | - | - | 3 |
| Mosaic trisomy 9 | 99776 | - | - | 2 |
| Mowat-Wilson syndrome | 2152 | 235730 | - | 10 |
| Muckle-Wells syndrome | 575 | 191900 | - | 1 |
| Mucolipidosis type II | 576 | 252500 | - | 1 |
| Mucopolysaccharidosis type 2 | 580 | 309900 | - | 12 |
| Mucopolysaccharidosis type 3 | 581 | 252900 | 252920 | 3 |
| Mucopolysaccharidosis type 6 | 583 | 253200 | - | 1 |
| Muenke syndrome | 53271 | 602849 | - | 3 |
| Multifocal lymphangioendotheliomatosis-thrombocytopenia syndrome | 464321 | - | - | 1 |
| Multiple congenital anomalies-hypotonia-seizures syndrome type 2 | 300496 | 300868 | - | 1 |
| Multiple endocrine neoplasia type 2A | 247698 | 171400 | - | 3 |
| Multiple endocrine neoplasia type 2B | 247709 | 162300 | - | 3 |
| Multiple epiphyseal dysplasia | 251 | - | - | 6 |
| Multiple osteochondromas | 321 | 133700 | 133701 | 2 |
| Muscle-eye-brain disease | 588 | 236670 | 253280 | 1 |
| Muscular pseudohypertrophy-hypothyroidism syndrome | 2349 | - | - | 1 |
| Myelomeningocele | 93969 | - | - | 8 |
| Myhre syndrome | 2588 | 139210 | - | 2 |
| Myoclonic-astastic epilepsy | 1942 | 615369 | 616421 | 12 |
| Nager syndrome | 245 | 154400 | - | 6 |
| Nail-patella syndrome | 2614 | 161200 | - | 15 |
| Nance-Horan syndrome | 627 | 302350 | - | 1 |
| Necrotizing enterocolitis | 391673 | - | - | 14 |
| Neonatal hemochromatosis | 446 | 231100 | - | 2 |
| Neonatal hypoxic and ischemic brain injury | 137577 | - | - | 8 |
| Neonatal Marfan syndrome | 284979 | - | - | 1 |
| Nephroblastoma | 654 | 194070 | 194071 | 41 |
| Nephrogenic diabetes insipidus | 223 | 125800 | 304800 | 3 |
| Netherton syndrome | 634 | 256500 | - | 3 |
| Neuroblastoma | 635 | 256700 | 613013 | 80 |
| Neurocutaneous melanocytosis | 2481 | 249400 | - | 2 |
| Neurodevelopmental disorder-craniofacial dysmorphism-cardiac defect-hip dysplasia syndrome due to a point mutation | 453504 | 616580 | - | 1 |
| Neuroendocrine cell hyperplasia of infancy | 217560 | - | - | 1 |
| Neurofibromatosis type 1 | 636 | 162200 | 162210 | 43 |
| Neurofibromatosis-Noonan syndrome | 638 | 601321 | - | 1 |
| Niemann-Pick disease type A | 77292 | 257200 | - | 1 |
| Niemann-Pick disease type C, juvenile neurologic onset | 216981 | - | - | 5 |
| Nijmegen breakage syndrome | 647 | 251260 | - | 1 |
| NLRP12-associated hereditary periodic fever syndrome | 247868 | 611762 | - | 1 |
| Noma | 2700 | - | - | 2 |
| Non-immune hydrops fetalis | 363999 | 236750 | - | 1 |
| Noonan syndrome | 648 | 163950 | 605275 | 60 |
| Norrie disease | 649 | 310600 | - | 2 |
| Oculocerebrorenal syndrome of Lowe | 534 | 309000 | - | 9 |
| Oculocutaneous albinism | 55 | - | - | 1 |
| Oculocutaneous albinism type 2 | 79432 | 203200 | - | 1 |
| Oculodentodigital dysplasia | 2710 | 164200 | 257850 | 2 |
| Oculofaciocardiodental syndrome | 2712 | 300166 | - | 2 |
| Odontochondrodysplasia | 166272 | 184260 | - | 1 |
| Ogden syndrome | 276432 | 300855 | - | 1 |
| Okamoto syndrome | 2729 | 604916 | - | 2 |
| Okihiro syndrome | 93293 | 607323 | - | 2 |
| Oligodontia | 99798 | 106600 | 150400 | 1 |
| Ollier disease | 296 | 166000 | - | 11 |
| Omphalocele | 660 | 164750 | 310980 | 29 |
| Ondine syndrome | 661 | 209880 | - | 7 |
| Opitz G/BBB syndrome | 2745 | 145410 | 300000 | 5 |
| Opsoclonus-myoclonus syndrome | 1183 | - | - | 7 |
| Optic atrophy-intellectual disability syndrome | 401777 | 615722 | - | 1 |
| Orofaciodigital syndrome type 1 | 2750 | 311200 | - | 4 |
| Osgood-Schlatter disease | 97335 | - | - | 10 |
| Osteofibrous dysplasia | 488265 | 607278 | - | 1 |
| Osteogenesis imperfecta type 1 | 216796 | 166200 | 166230 | 5 |
| Osteogenesis imperfecta type 3 | 216812 | 259420 | 259440 | 1 |
| Osteopathia striata-cranial sclerosis syndrome | 2780 | 300373 | - | 2 |
| Osteosarcoma | 668 | 259500 | - | 51 |
| Otomandibular syndrome | 141136 | 164210 | - | 5 |
| Pachydermoperiostosis | 2796 | 167100 | 259100 | 1 |
| PANDAS | 66624 | - | - | 63 |
| Panner disease | 97336 | - | - | 1 |
| Parkes Weber syndrome | 90307 | 608354 | 608355 | 2 |
| Paroxysmal dyskinesia | 1431 | - | - | 8 |
| Paroxysmal extreme pain disorder | 46348 | 167400 | - | 2 |
| Pearson syndrome | 699 | 557000 | - | 1 |
| Pediatric hepatocellular carcinoma | 33402 | - | - | 1 |
| Pediatric multiple sclerosis | 477738 | - | - | 3 |
| Pendred syndrome | 705 | 274600 | - | 5 |
| Pentalogy of Cantrell | 1335 | 313850 | - | 1 |
| Perlman syndrome | 2849 | 267000 | - | 1 |
| Peroxisome biogenesis disorder | 79189 | - | - | 1 |
| Persistent hyperplastic primary vitreous | 91495 | 221900 | 611308 | 1 |
| Peters anomaly | 708 | 604229 | 612968 | 5 |
| Peters plus syndrome | 709 | 261540 | - | 2 |
| PFAPA syndrome | 42642 | - | - | 6 |
| PHACE syndrome | 42775 | 606519 | - | 7 |
| Phenylketonuria | 716 | 261600 | - | 72 |
| Piebaldism | 2884 | 172800 | - | 8 |
| Pierpont syndrome | 487825 | 602342 | - | 1 |
| Pili torti | 2889 | 261900 | - | 1 |
| Pitt-Hopkins syndrome | 2896 | 610954 | - | 7 |
| Pitt-Hopkins-like syndrome | 221150 | 610042 | 614325 | 2 |
| Pituitary stalk interruption syndrome | 95496 | - | - | 1 |
| Pityriasis rubra pilaris | 2897 | 173200 | - | 4 |
| Pleuropulmonary blastoma | 64742 | 601200 | - | 1 |
| Poland syndrome | 2911 | 173800 | - | 34 |
| Poliomyelitis | 2912 | - | - | 7 |
| Polymicrogyria | 35981 | - | - | 26 |
| Pontine tegmental cap dysplasia | 269229 | 614688 | - | 1 |
| Pontocerebellar hypoplasia type 2 | 2524 | 277470 | 612389 | 2 |
| Popliteal pterygium syndrome | 294963 | - | - | 1 |
| Porencephaly | 2940 | 175780 | 614483 | 1 |
| Porphyria due to ALA dehydratase deficiency | 100924 | 612740 | - | 1 |
| Port-wine nevi-mega cisterna magna-hydrocephalus syndrome | 2703 | - | - | 2 |
| Postaxial polydactyly-anterior pituitary anomalies-facial dysmorphism syndrome | 420584 | 615849 | - | 1 |
| Posterior urethral valve | 93110 | - | - | 11 |
| Potocki-Shaffer syndrome | 52022 | 601224 | - | 1 |
| Prader-Willi syndrome | 739 | 176270 | 615547 | 82 |
| Prader-Willi syndrome due to a point mutation | 398069 | 615547 | - | 1 |
| Precocious puberty | 95708 | - | - | 7 |
| Primary ciliary dyskinesia | 244 | 215518 | 215520 | 6 |
| Progressive familial intrahepatic cholestasis | 172 | 211600 | 601847 | 3 |
| Progressive osseous heteroplasia | 2762 | 166350 | - | 1 |
| Propionic acidemia | 35 | 606054 | - | 3 |
| Proteus syndrome | 744 | 176920 | - | 3 |
| Proximal spinal muscular atrophy type 1 | 83330 | 253300 | - | 24 |
| Proximal spinal muscular atrophy type 2 | 83418 | 253550 | - | 6 |
| Proximal spinal muscular atrophy type 3 | 83419 | 253400 | - | 5 |
| Prune belly syndrome | 2970 | 100100 | - | 20 |
| Pseudoachondroplasia | 750 | 177170 | - | 3 |
| Pseudohypoparathyroidism with Albright hereditary osteodystrophy | 457059 | - | - | 1 |
| Pulmonary interstitial glycogenosis | 217557 | - | - | 1 |
| Pulmonary valve agenesis | 982 | - | - | 1 |
| Pyridoxine-dependent epilepsy | 3006 | 266100 | 617290 | 2 |
| Pyruvate carboxylase deficiency | 3008 | 266150 | - | 1 |
| Pyruvate dehydrogenase deficiency | 765 | 245348 | 245349 | 6 |
| Radial hemimelia | 93321 | - | - | 3 |
| Rapid-onset childhood obesity-hypothalamic dysfunction-hypoventilation-autonomic dysregulation syndrome | 293987 | - | - | 9 |
| Rare congenital anomaly of ventricular septum | 474347 | - | - | 1 |
| Rare lymphatic malformation | 2415 | - | - | 3 |
| Recessive X-linked ichthyosis | 461 | 300001 | 308100 | 1 |
| Recombinant 8 syndrome | 96167 | 179613 | - | 2 |
| Reflex epilepsy | 310 | - | - | 1 |
| Regional odontodysplasia | 83450 | - | - | 1 |
| Reis-Bücklers corneal dystrophy | 98961 | 608470 | - | 1 |
| Renal agenesis | 411709 | 191830 | 615721 | 2 |
| Renal agenesis, bilateral | 1848 | 191830 | 617805 | 2 |
| Renal or urinary tract malformation | 93545 | - | - | 1 |
| Renpenning syndrome | 3242 | 309500 | - | 1 |
| Retinoblastoma | 790 | 180200 | - | 89 |
| Retinopathy of prematurity | 90050 | 133780 | - | 13 |
| Rett syndrome | 778 | 312750 | - | 81 |
| Rhabdoid tumor | 69077 | 609322 | 613325 | 5 |
| Rhabdomyosarcoma | 780 | 268210 | 268220 | 33 |
| Rheumatic fever | 3099 | 268240 | - | 18 |
| Rhizomelic chondrodysplasia punctata | 177 | 215100 | 222765 | 2 |
| Rhombencephalosynapsis | 59315 | - | - | 3 |
| Riboflavin transporter deficiency | 97229 | 211500 | 211530 | 2 |
| Ring chromosome 13 syndrome | 96176 | - | - | 1 |
| Ring chromosome 14 syndrome | 1440 | 616606 | - | 3 |
| Ring chromosome 15 syndrome | 96177 | - | - | 1 |
| Ring chromosome 18 syndrome | 1442 | - | - | 3 |
| Ring chromosome 20 syndrome | 1444 | - | - | 3 |
| Ring chromosome 4 syndrome | 1447 | - | - | 1 |
| Ring chromosome 8 syndrome | 1450 | - | - | 1 |
| Ring chromosome 9 syndrome | 96173 | - | - | 1 |
| Robinow syndrome | 97360 | 180700 | 268310 | 3 |
| Roifman syndrome | 353298 | 300258 | 616651 | 1 |
| Rolandic epilepsy | 1945 | 117100 | 245570 | 4 |
| Rothmund-Thomson syndrome | 2909 | 268400 | - | 4 |
| Rubinstein-Taybi syndrome | 783 | 180849 | 610543 | 20 |
| Saethre-Chotzen syndrome | 794 | 101400 | - | 8 |
| Salla disease | 309334 | 604369 | - | 2 |
| Salt-and-pepper syndrome | 370938 | 609056 | - | 1 |
| Sandhoff disease, infantile form | 309155 | 268800 | - | 3 |
| Sanfilippo syndrome type B | 79270 | 252920 | - | 1 |
| Scapuloperoneal spinal muscular atrophy | 431255 | 181405 | - | 1 |
| Schimke immuno-osseous dysplasia | 1830 | 242900 | - | 1 |
| Schinzel-Giedion syndrome | 798 | 269150 | - | 1 |
| Schizencephaly | 799 | 269160 | - | 32 |
| Schwartz-Jampel syndrome | 800 | 255800 | - | 2 |
| Scimitar syndrome | 185 | - | - | 3 |
| Senior-Loken syndrome | 3156 | 266900 | 606995 | 1 |
| Septo-optic dysplasia spectrum | 3157 | 182230 | - | 19 |
| Severe combined immunodeficiency | 183660 | - | - | 18 |
| Severe combined immunodeficiency due to adenosine deaminase deficiency | 277 | 102700 | - | 3 |
| Severe congenital neutropenia | 42738 | - | - | 1 |
| Severe feeding difficulties-failure to thrive-microcephaly due to ASXL3 deficiency syndrome | 352577 | 615485 | - | 2 |
| Severe hemophilia A | 169802 | 306700 | - | 1 |
| Severe hemophilia B | 169793 | 306900 | - | 2 |
| Sheldon-Hall syndrome | 1147 | 601680 | 616266 | 1 |
| Short chain acyl-CoA dehydrogenase deficiency | 26792 | 201470 | - | 1 |
| Short rib-polydactyly syndrome | 1505 | - | - | 1 |
| Short stature-brachydactyly-obesity-global developmental delay syndrome | 464288 | 617157 | - | 1 |
| Shprintzen-Goldberg syndrome | 2462 | 182212 | - | 4 |
| Shwachman-Diamond syndrome | 811 | 260400 | 617941 | 14 |
| Silver-Russell syndrome | 813 | 180860 | 312780 | 27 |
| Simpson-Golabi-Behmel syndrome | 373 | 312870 | - | 1 |
| Sirenomelia | 3169 | 600145 | - | 3 |
| Sjögren-Larsson syndrome | 816 | 270200 | - | 1 |
| SLC35A2-CDG | 356961 | 300896 | - | 1 |
| Smith-Lemli-Opitz syndrome | 818 | 270400 | - | 14 |
| Smith-Magenis syndrome | 819 | 182290 | - | 20 |
| Solitary median maxillary central incisor syndrome | 2286 | - | - | 1 |
| Sotos syndrome | 821 | 117550 | 617169 | 39 |
| Spasmus nutans | 279882 | - | - | 1 |
| Spastic paraplegia-intellectual disability-nystagmus-obesity syndrome | 521390 | 617296 | - | 1 |
| Spinocerebellar ataxia type 29 | 208513 | 117360 | - | 1 |
| Spondyloepiphyseal dysplasia congenita | 94068 | 183900 | - | 1 |
| Steel syndrome | 438117 | 615155 | - | 1 |
| Stickler syndrome | 828 | 108300 | 604841 | 22 |
| Stromme syndrome | 506307 | 243605 | - | 1 |
| Sturge-Weber syndrome | 3205 | 185300 | - | 15 |
| Succinic semialdehyde dehydrogenase deficiency | 22 | 271980 | - | 1 |
| Syndromic diarrhea | 84064 | 222470 | 614602 | 1 |
| Systemic-onset juvenile idiopathic arthritis | 85414 | 604302 | - | 17 |
| Tall stature-intellectual disability-facial dysmorphism syndrome | 404443 | 615879 | - | 1 |
| Tangier disease | 31150 | 205400 | - | 2 |
| TARP syndrome | 2886 | 311900 | - | 1 |
| Temple syndrome due to maternal uniparental disomy of chromosome 14 | 96184 | 616222 | - | 1 |
| Tessier number 7 facial cleft | 141276 | 613545 | - | 1 |
| Testicular regression syndrome | 983 | 273250 | - | 1 |
| Tetragametic chimerism | 199310 | - | - | 1 |
| Tetralogy of Fallot | 3303 | 187500 | - | 86 |
| Tetrasomy 12p | 884 | 601803 | - | 3 |
| Tetrasomy 18p | 3307 | 614290 | - | 5 |
| Tetrasomy 9p | 3310 | - | - | 2 |
| Tetrasomy X | 9 | - | - | 4 |
| Thanatophoric dysplasia | 2655 | 156830 | - | 5 |
| Thiamine-responsive megaloblastic anemia syndrome | 49827 | 249270 | - | 1 |
| Thrombocytopenia-absent radius syndrome | 3320 | 274000 | - | 8 |
| Tibial hemimelia | 93322 | 275220 | - | 3 |
| Toriello-Carey syndrome | 3338 | 217980 | - | 1 |
| Toxocariasis | 3343 | - | - | 1 |
| Tracheal agenesis | 3346 | - | - | 1 |
| Transient erythroblastopenia of childhood | 98871 | 227050 | - | 1 |
| Transposition of the great arteries | 216675 | - | - | 18 |
| Treacher-Collins syndrome | 861 | 154500 | 248390 | 14 |
| Tricho-dento-osseous syndrome | 3352 | 190320 | - | 1 |
| Trichorhinophalangeal syndrome type 2 | 502 | 150230 | - | 2 |
| Trichothiodystrophy | 33364 | 234050 | 300953 | 1 |
| Tricuspid atresia | 1209 | 605067 | - | 12 |
| Triploidy | 3376 | - | - | 5 |
| Trisomy 12p | 1699 | - | - | 1 |
| Trisomy 13 | 3378 | - | - | 52 |
| Trisomy 18 | 3380 | - | - | 84 |
| Trisomy 1q | 261344 | - | - | 1 |
| Trisomy 20p | 261318 | - | - | 1 |
| Trisomy 4p | 1738 | - | - | 1 |
| Trisomy 5p | 1742 | - | - | 2 |
| Trisomy 8p | 264450 | - | - | 1 |
| Trisomy 9p | 236 | - | - | 3 |
| Trisomy X | 3375 | - | - | 19 |
| Tritanopia | 88629 | 190900 | - | 1 |
| Truncus arteriosus | 3384 | 217095 | - | 17 |
| Tufted angioma | 1063 | 607859 | - | 1 |
| Turner syndrome | 881 | - | - | 97 |
| Ulnar hemimelia | 93320 | - | - | 1 |
| Ulnar-mammary syndrome | 3138 | 181450 | - | 1 |
| Uncombable hair syndrome | 1410 | 191480 | 617251 | 2 |
| Univentricular heart | 1464 | - | - | 2 |
| Unverricht-Lundborg disease | 308 | 254800 | 310370 | 3 |
| Usher syndrome | 886 | 276900 | 276901 | 24 |
| Usher syndrome type 1 | 231169 | 276900 | 276904 | 1 |
| VACTERL/VATER association | 887 | 192350 | - | 15 |
| Van der Woude syndrome | 888 | 119300 | 604547 | 1 |
| Vein of Galen aneurysmal malformation | 1053 | 618196 | - | 3 |
| Vernal keratoconjunctivitis | 70476 | - | - | 2 |
| Very long chain acyl-CoA dehydrogenase deficiency | 26793 | 201475 | - | 9 |
| Vici syndrome | 1493 | 242840 | - | 1 |
| Waardenburg syndrome | 3440 | 148820 | 193500 | 6 |
| Wagner disease | 898 | 143200 | - | 1 |
| WAGR syndrome | 893 | 194072 | 612469 | 2 |
| Walker-Warburg syndrome | 899 | 236670 | 253280 | 1 |
| Weaver syndrome | 3447 | 277590 | - | 3 |
| West syndrome | 3451 | 300672 | 308350 | 66 |
| Wiedemann-Rautenstrauch syndrome | 3455 | 264090 | - | 2 |
| Wiedemann-Steiner syndrome | 319182 | 605130 | - | 4 |
| Wildervanck syndrome | 3456 | 314600 | - | 1 |
| Williams syndrome | 904 | 194050 | - | 87 |
| Wilson disease | 905 | 277900 | - | 43 |
| Wilson-Turner syndrome | 3459 | 309585 | - | 1 |
| Wiskott-Aldrich syndrome | 906 | 301000 | 600903 | 9 |
| Wolf-Hirschhorn syndrome | 280 | 194190 | - | 14 |
| Wolman disease | 75233 | 278000 | - | 1 |
| Worster-Drought syndrome | 3465 | 185480 | - | 2 |
| Wyburn-Mason syndrome | 53719 | - | - | 1 |
| X-linked agammaglobulinemia | 47 | 300310 | 300755 | 2 |
| X-linked centronuclear myopathy | 596 | 310400 | - | 1 |
| X-linked cone dysfunction syndrome with myopia | 90001 | 300843 | - | 2 |
| X-linked creatine transporter deficiency | 52503 | 300352 | - | 1 |
| X-linked hypophosphatemia | 89936 | 307800 | - | 11 |
| X-linked intellectual disability, Najm type | 163937 | 300749 | - | 1 |
| X-linked intellectual disability, Snyder type | 3063 | 309583 | - | 1 |
| X-linked retinoschisis | 792 | 312700 | - | 4 |
| Xp22.13p22.2 duplication syndrome | 284180 | - | - | 4 |
