# Supplementary figures and images for: Facebook Support Groups for Rare Pediatric Diseases: Quantitative Analysis
Source: JMIR Pediatr Parent. 2020 Nov 19;3(2):e21694. doi: 10.2196/21694 (PMC7714646; doi:10.2196/21694)

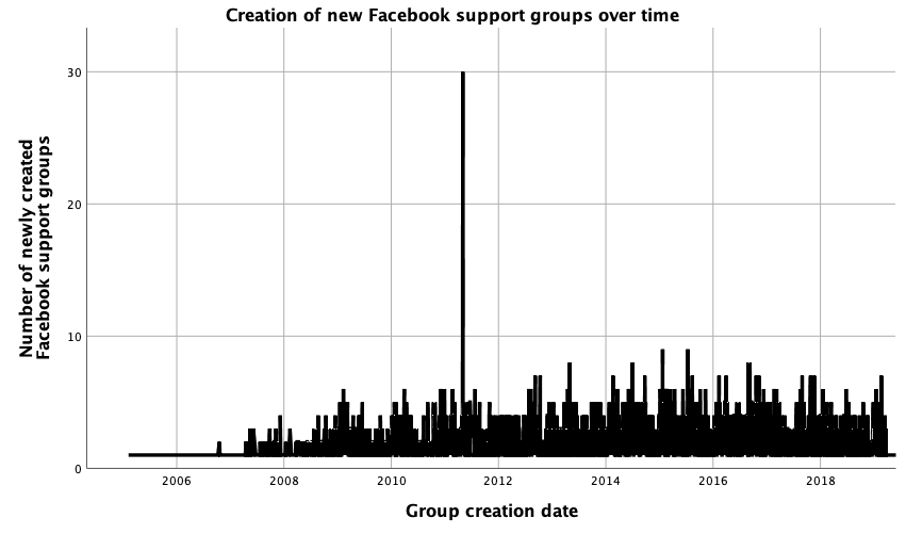

Supplement: Multimedia Appendix 2 [file pediatrics_v3i2e21694_app2.png]
