# Supplementary material for: Facebook Support Groups for Rare Pediatric Diseases: Quantitative Analysis
Source: JMIR Pediatr Parent. 2020 Nov 19;3(2):e21694. doi: 10.2196/21694 (PMC7714646; doi:10.2196/21694)
Supplement: Multimedia Appendix 3 [file pediatrics_v3i2e21694_app3.doc]

***Multimedia Appendix 3. Full list of number of Facebook support groups by language.***

| **Language** | **Frequency** | **Percent** |
| --- | --- | --- |
| English | 5721 | 89,4 |
| French | 227 | 3,5 |
| Spanish | 99 | 1,5 |
| German | 42 | ,7 |
| Dutch | 28 | ,4 |
| Portuguese | 27 | ,4 |
| Swedish | 26 | ,4 |
| Turkish | 26 | ,4 |
| Polish | 24 | ,4 |
| Danish | 23 | ,4 |
| Italian | 22 | ,3 |
| Arabic | 13 | ,2 |
| Russian | 12 | ,2 |
| Hungarian | 11 | ,2 |
| Chinese | 10 | ,2 |
| Norwegian | 9 | ,1 |
| Czech | 8 | ,1 |
| Hebrew | 8 | ,1 |
| Finnish | 7 | ,1 |
| Greek | 7 | ,1 |
| Japanese | 7 | ,1 |
| Indonesian | 6 | ,1 |
| Malaysian | 4 | ,1 |
| Thai | 4 | ,1 |
| Afrikaans | 3 | ,0 |
| Romanian | 3 | ,0 |
| Vietnamese | 3 | ,0 |
| Bosnian | 2 | ,0 |
| Bulgarian | 2 | ,0 |
| Croatian | 2 | ,0 |
| Filipino | 2 | ,0 |
| Icelandic | 2 | ,0 |
| Aserbaidshan | 1 | ,0 |
| Persian | 1 | ,0 |
| Serbian | 1 | ,0 |
| Slovakian | 1 | ,0 |
| Slovenian | 1 | ,0 |
| Urdu | 1 | ,0 |
| unknown | 2 | ,0 |
| Total | 6398 | 100,0 |
